# Retraction Note: The Adipocyte Na/K-ATPase Oxidant Amplification Loop is the Central Regulator of Western Diet-Induced Obesity and Associated Comorbidities

**DOI:** 10.1038/s41598-022-13459-9

**Published:** 2022-05-30

**Authors:** Rebecca D. Pratt, Cameron Brickman, Athar Nawab, Cameron Cottrill, Brian Snoad, Hari Vishal Lakhani, Austin Jelcick, Brandon Henderson, Niharika N. Bhardwaj, Juan R. Sanabria, Jiang Liu, Zijian Xie, Nader G. Abraham, Joseph I. Shapiro, Komal Sodhi

**Affiliations:** 1 Departments of Medicine, Surgery, Biomedical Sciences, and Healthcare Informatics Program, Joan C. Edwards School of Medicine, Marshall University, Huntington, WV USA; 2 Vector Builder Incorporated, Shenandoah, TX 77384 USA; 3 Department of Medicine, New York Medical College, Valhalla, NY 10595 USA

Retraction of: *Scientific Reports* 10.1038/s41598-019-44350-9, published online 28 May 2019

The Editors have retracted this Article.

After publication, concerns were raised regarding high similarity among different lanes in the Coomassie blue staining image in Fig. 4d. Further investigation has found that there are inconsistencies in the blot backgrounds in Figs. S5d, g and f, and S6b, d and f, which undermine the integrity of these gels. Furthermore, in the raw data provided by the authors (links to depository in the Data Availability section), there are unexplained similarities between images representing brain tissue "control - no treatment" and "wd - no treatment" groups (dapi channel; rotated by 180 degrees), and between images of liver tissue “wd-lenti-adipo-naktide” and “wd-lenti-adipo-gfp” groups (dapi channel; gfp image appears to be stretched).

The Editors therefore no longer have confidence in the presented data.

Brandon Henderson, Juan R. Sanabria, and Nader G. Abraham agree to this retraction. Rebecca D. Pratt, Cameron Cottrill, Niharika N. Bhardwaj, Jiang Liu, Joseph I. Shapiro, and Komal Sodhi do not agree to this retraction. Cameron Brickman, Athar Nawab, Brian Snoad, Hari Vishal Lakhani, Austin Jelcick, and Zijian Xie have not responded to any correspondence from the Editors about this retraction.

